# Nutritional Programming: History, Hypotheses, and the Role of Prenatal Factors in the Prevention of Metabolic Diseases—A Narrative Review

**DOI:** 10.3390/nu14204422

**Published:** 2022-10-21

**Authors:** Izabela Michońska, Edyta Łuszczki, Magdalena Zielińska, Łukasz Oleksy, Artur Stolarczyk, Katarzyna Dereń

**Affiliations:** 1 Institute of Health Sciences, College of Medical Sciences, University of Rzeszow, 35-959 Rzeszow, Poland; 2 Faculty of Health Sciences, Department of Physiotherapy, Jagiellonian University Medical College Krakow, 31-008 Krakow, Poland; 3 Orthopedic and Rehabilitation Department, Medical Faculty, Medical University of Warsaw, 02-091 Warsaw, Poland

**Keywords:** nutritional programming, nutrition, obesity, children

## Abstract

Childhood obesity and the numerous lifestyle diseases associated with it are undoubtedly among the key problems in modern medicine and public health. However, this problem concerns not only the present or immediate future, but also the longer term. Adult health is fundamentally shaped in the first years of life and in the fetal period. The preconceptual period, which is responsible for the proper preparation of the internal environment for the life and development of the fetus during pregnancy, is also significant. A special role in describing the phenomenon of conditioning the metabolism of the new human being is now attributed to the theory of nutritional programming. Research in this area was pioneered by David Barker, who put forward the theory of the “stunted phenotype” and described the relationship between a child’s birth weight, which is largely a consequence of the mother’s feeding behaviour, and diseases such as ischaemic heart disease, type 2 diabetes (T2D), dyslipidemia, or high blood pressure. This narrative review aims to provide an overview of the history, theory, and prenatal mechanisms involved in nutritional programming and its relationship to childhood obesity and other metabolic diseases.

## 1. Introduction

The term nutritional programming was introduced into medical literature by the British physician and epidemiologist David Barker more than four decades ago [1]. This concept assumes that during human fetal development, the so-called critical periods (critical windows) are distinguished [1,2,3]. During these periods, various metabolic and hormonal changes may occur that affect health later in life. Epidemiological factors include the impact of adverse environmental stimuli, such as deficiency or excess of a particular nutrient, and the resulting irreversible structural, metabolic and functional damage to individual organs of the child [2,3]. This process is associated with the risk of numerous metabolic diseases known as ‘civilization diseases’, including type 2 diabetes (T2D), hyperlipidemia, obesity, osteoporosis, high blood pressure, cancer and cardiovascular diseases [3,4,5].

The focus of this article is strictly on prenatal issues and the importance of the preconceptional period in the context of nutritional programming. Postnatal components are an equally important determinant of metabolic health, but are not the focus of this paper, due to the breadth of the issue and the large number of papers [6,7,8,9,10,11,12,13,14,15,16,17,18,19]. It should be mentioned that recent studies point to the importance of breastfeeding at birth, including comparison to formula feeding, and the risk of developing obesity in later years of life, pointing, among other things, to the mechanisms of regulation of satiety [7,8,9,10,11,12,13,14,15,16]. Studies also point to the problem of excess protein in a child’s diet or the role of normal body iron metabolism [17,18,19]. The factors most often considered in fetal life are too high or too low energy availability, as well as maternal body composition [13,14,15]. Other components include maternal obesity, gestational diabetes mellitus (GDM), maternal diet, including fat supply, or the role of various factors in shaping the child’s gut microbiota [20,21,22,23,24,25,26,27,28,29,30,31,32,33].

Part of the explanation for the increase in the incidence of metabolic disorders in children at increasingly earlier stages of life may therefore be the lack of interdisciplinary care or adequate preventive programmes for women of reproductive age in some developing countries [34,35,36]. A (worldwide) solution to this problem could be to provide women planning a pregnancy with universal access to nutrition education during the preconception, pregnancy and lactation periods, along the lines of programmes such as The Special Supplemental Nutrition Program for Women, Infants, and Children (WIC), Nutrition education and counselling (NEC) or Nutritional Intervention Preconception and During Pregnancy to Maintain Healthy Glucose Metabolism and Offspring Health (NiPPeR) [35,37,38]. WIC is a program conducted in the US, NiPPeR was a study conducted in 3 countries, the United Kingdom (UK), Singapore and New Zealand, while NEC interventions have been conducted in many countries around the world with different levels of development (low, middle and high income countries) including China, Greece, India, Nepal and Egypt [35,37,38].

## 2. Methods

Research was conducted by using PubMed/MEDLINE, Cochrane Library, Science Direct, MEDLINE, and EBSCO databases, from July 2022 to August 2022, for English language meta-analyses, systematic reviews, randomized clinical trials, and observational studies from all over the world. The websites of scientific organizations, such as WHO, were also searched. Eight main topics were defined: (1) the history of nutritional programming; (2) the thrifty phenotype hypothesis; (3) the ‘fetal salvage’ hypothesis; (4) the ‘catch-up growth hypothesis’; (5) epigenetic/DNA methylation and posttranslational modification of histones; (6) obesity; (7) gestational diabetes; (8) gut microbiota.

Inclusion criteria: articles in English; nutritional programming and prenatal issues; nutritional programming and preconception period; meta-analyses; systematic reviews; randomized clinical trials; observational studies; animal studies; historical data. Exclusion criteria: articles in a language other than English; nutritional programming and postnatal issues (breastfeeding, protein intake); in vitro studies, book, narrative reviews, research with pregnant adolescents (see Table 1).

## 3. Nutritional Programming

### 3.1. History

The importance of proper nutrition for both pre-conceptional and pregnant women was emphasized as early as 1930–1939. The beginning of research on this topic is credited to the English pharmacist Edward Mellandby [39]. He was one of the first scientists to even report links between fetal health and the mother’s eating habits, even those of her childhood or the diet of earlier generations, in his speculations. He pointed out the role of an adequate supply of calcium, vitamin D, or iodine during pregnancy to avoid diseases or symptoms such as rickets or simple goitre [39].

Concrete concept of nutritional programming was first introduced into medical terminology in the 1980s by David Barker. He was a British physician and epidemiologist and a renowned scientist associated with the University of Southampton. He conducted extensive research on metabolism and the link between fetal conditions and the incidence of disease later in life [1,4,5]. He and other researchers observed that, despite the increasing wealth of societies, not only the wealthier residents of each country suffer from cardiovascular disease. Numerous groups of people of lower economic status, often at risk of prenatal malnutrition, were also affected. This indicated the importance of fetal exposure to adverse living conditions and their relationship with health outcomes later in life. [40,41,42,43]. All the researchers who presented their hypotheses in the 1980s relied on numerous observational studies conducted independently by researchers in many countries, including: The UK, Sweden, USA, Finland or China [40,41,42,43,44,45,46,47,48,49]. The Overkalix cohort was a study often cited in the context of inheritance and the link between ancestral nutritional status and the health of future generations, and this link was particularly emphasized for male descendants [45]. In contrast, more recent studies that have attempted to replicate the conditions of the cohort have failed to confirm an unequivocal link with mortality from diabetes or cardiovascular disease, mainly due to a significant increase in cancer mortality [45,50,51]. However, the skeptics saw some limitations in the methodology of these studies. First, they were cohort or observational studies with numerous confounding factors, such as lack of social class division or conducting research primarily with men [52]. Nevertheless, the study’s authors report that for some of the men, they had information on social class and that their birth weight was not related to social class [43]. Second, the results of the studies were only related to the low birth weight of the newborns, which, according to some, did not justify being considered reliable in the context of nutritional programming, since low birth weight can be a consequence of many variables [20,52,53].

A breakthrough finding, which gives reason to link it to nutrition programming theory, was the Dutch Hunger Winter cohort conducted in the offspring born during or after the so-called Dutch famine, which lasted from 1944 to 1945 in the western parts of the Netherlands [54,55,56]. This famine, which lasted more than six months, was the result of the occupation of the Netherlands by the German Reich and affected about 4.5 million Dutch people. The death toll from this event is estimated to be between 18,000 and 29,000, depending on the source, and it is reported that these were mainly elderly men [54]. However, food shortages and 400–800 kcal/day rations also affected pregnant women and young children. After the war, economic conditions improved and access to food increased. The results of this study, conducted in Amsterdam at the end of the war, were only revealed in 1979, more than 30 years after the famine winter. The following tragic events of famine and years of fertility were thus almost a first-class human experiment, which no one would ever have authorized on bioethical grounds [55]. The main conclusion of this experiment was to link fetus exposure to harsh intrauterine conditions with its health status in adulthood. However, it was not always associated with intrauterine growth restriction (IUGR), which signifies too low a body weight for age and can also result in low birth weight [56,57].

In the 21st century, many new studies have emerged on intrauterine programming and the relevance of the child’s diet during the first two years of life. New hypotheses have also been published, as described below.

### 3.2. Hypotheses

**Figure 1:**
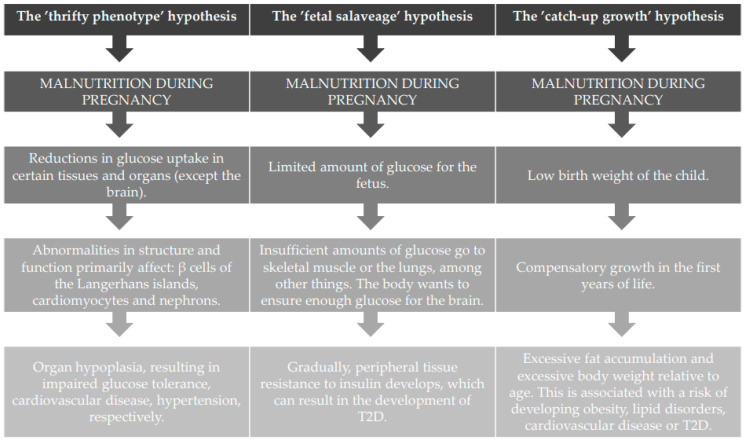
Three main hypotheses for nutrition programming.

There are three main hypotheses for nutrition programming, which are described in detail below and summarized in the figure (see Figure 1).

#### 3.2.1. The Thrifty Phenotype Hypothesis

The Barker hypothesis, published in 1990, identified the group most at risk of metabolic complications as those who had experienced conditions that were unfavorable to normal development during fetal life and who had unrestricted access to food in adulthood [1,4]. It is now also often referred to as the thrifty phenotype hypothesis [58]. In a study published in 1989, Barker et al. described the association of a child’s birth weight with the possibility of developing ischaemic heart disease [43]. The trial involved an analysis of 5654 men born in England between 1911 and 1930. The study’s conclusions were that the lower the birth weight of the child and the lower the increase in weight in the first year of life, the higher the mortality rate from coronary heart disease [43]. Another study showed that higher systolic blood pressure was also associated with low birth weight in men [59]. The conclusions reached by the researchers spoke of the need for growth promoters during pregnancy and in the first years of a child’s life [41,42,43].

It is worth mentioning that studies conducted on the relationship between exposure to malnutrition during pregnancy (affecting, among other things, fetal conditions or child birth weight) and the development of diseases have been conducted exclusively with men [45,59]. However, men were often the group in which the incidence of cardiovascular disease or hypertension was more frequent or had more serious consequences [45,59]. However, certain biological factors, such as endogenous sex hormones, type of work, body weight, or habits about stimulants (cigarettes, alcohol) can play a protective role in women [59]. Recent studies confirm, among other things, the protective effect of estrogen in the context of cardiovascular disease, as well [60,61]. In multi-year cohort studies, men are also easier to track down years later because they rarely change their surnames [43].

Subsequent research conducted by Professor Barker, though also by other researchers was related to the relationship between birth weight and the incidence of diseases such as type 2 diabetes, dyslipidemia, hypertension, and stroke [5,41,43,58,59,62,63]. The results of his study and those of many other researchers have finally made it possible to describe the three basic structures of the fetal body that are most affected by malnutrition during pregnancy [4,5,40,41,42,43,46,47,58,59,62,63]. Abnormalities in structure and function primarily affect: the β-cells of the Langerhans islets, cardiomyocytes, and nephrons cells [58]. With limited availability of food in fetal life, all of the above changes are necessary for the normal development of the most important organ—the brain [58,62]. Restricted access to glucose in the pancreas, kidneys, or heart results in a reduction in the number of cell divisions in these organs, resulting in hypoplasia and a decrease in their performance [5,58,59]. As the body matures and reaches adulthood, the less functioning organs are more prone to develop further disorders [58,62,64]. Therefore, the pancreas develops a progressive impairment of glucose tolerance and type 2 diabetes in adulthood [58,62]. A low number of kidney nephrons predispose hypertension, and a less developed heart is more prone to coronary heart disease or myocardial infarction [4,5,58,62,64].

#### 3.2.2. The ‘Fetal Salvage’ Hypothesis

Another theory of intrauterine programming is the fetal salvage hypothesis [65]. This was announced by Hofman et al. in 1997 on the basis of a study, which reported that due to maternal malnutrition, the fetus receives only limited amounts of glucose [65,66,67,68,69,70,71]. For this reason, a malnourished fetus develops successive peripheral insulin resistance and available glucose is diverted to the brain, at the expense of skeletal muscle or the lungs [65,66,71]. Reduced skeletal muscle results in a worsening of body glucose metabolism; pancreatic β-cells are stimulated to increase insulin production to achieve normoglycemia [65,66]. The long-term exploitation of pancreatic islet cells over time must lead to the development of serious diseases [65,66,71]. It should be noted that, according to this hypothesis, cases where a child is diagnosed with IUGR can be considered a putative marker of the development of type 2 diabetes [65,71]. In addition, the severity of birth hypotrophy has been associated with reduced insulin reserves in adults [72].

Both hypotheses presented above are related to the existence of so-called critical moments of fetal development [1,2,3,58,65]. If malnutrition occurs at key moments of life, including the formation of human cells, tissues, or organs and the programming of their functions, the effects on metabolism may be very serious and even irreversible [1,2,3,4,5,41,43,58,65,66,67,68,69,70,71,72]. A solution to these problems can be provided by properly designed and implemented nutritional therapy for women of reproductive age and pregnant women [1,2,3].

#### 3.2.3. The ‘Catch-Up Growth’ Hypothesis

In addition to theories of fetal life, there is also the “catch-up growth” hypothesis, which is also indirectly related to fetal life and too low birth weight [73,74,75,76]. It refers to the compensatory growth of the body in the first 2 years of life of children born with low birth weight [73,76]. This is because after months of struggle to survive, the child’s body experiences prosperity in the postnatal environment. The increased quality of life and virtually unlimited access to food in most modern societies is not without influence. This situation is undoubtedly also facilitated by the increasing number of fast-food outlets or the presence of ultra-processed foods in the global food supply [77,78,79,80,81]. In addition, highly processed foods have been indicated as very harmful to health, not just for children [78,79,80,81]. Excessive caloric intake and the high energy density of the meals consumed easily allow children born with hypotrophy to accelerate growth and increase weight, increase waist circumference, or increase total and LDL cholesterol [79,80,81]. Therefore, children with IUGR catch up with their counterparts born with normal body weight and who do not experience adaptations such as hypoplasia of the internal organs during fetal life. Postnatal weight compensation is often synonymous with increased body fat, as well as fluctuations in sensitivity to the hypoglycemic hormone insulin. Rapid compensatory measures can lead to the development of various diseases, including obesity, type 2 diabetes, hyperlipidemia, and CHS [76].

### 3.3. Preconceptive and Prenatal Factors in Nutritional Programming

#### 3.3.1. Epigenetics/DNA Methylation and Post-Translational Modification of Histones

Modern scientists are paying increasing attention to the exploration of epigenetics. The term has been around in medicine since 1939 and is loosely translated as “beyond genetics” [82]. Conrad H. Waddington used it to explain why embryonic stem cells differentiate into many different tissues despite having the same genetic material. Thus, this science covers problems of variation in gene expression, but does not include changes associated with the modification of nucleotide sequences in DNA [83]. Factors that influence gene expression variability are environmental factors, also known as external factors. From the point of view of nutritional programming, it is extremely important that these modified genes, which do not carry a change in DNA sequence, can also be inherited [82]. Currently, DNA methylation and post-translational modification of histones are cited as the two main mechanisms of epigenetic variation that can play a significant role in intrauterine programming [21,84]. It should be noted that changes at the epigenetic level associated with prenatal exposure to adverse environmental conditions and the resulting physiological or metabolic adaptations of the offspring may persist despite the cessation of the deleterious external agent [85].

DNA methylation is a type of epigenetic change that involves the covalent modification of the fifth carbon atom bound to cytosine. On the contrary, post-translational modification of histone proteins consists of methylation, phosphorylation, and acetylation, among others. Transformations at the epigenetic level of DNA and histones occur by increasing or decreasing DNA access to transcription machinery, both enhancers and repressors [84]. Both mechanisms of epigenetic variation mentioned above through cell divisions by mitosis and meiosis can pass into progeny cells. The epigenetic nature of variation is often associated with so-called critical periods in fetal development. During these periods, the “plasticity” of the organism is considered to be part of the adaptation to environmental conditions. However, in the early stages of life, it rarely manifests itself at the tissue level and, as researchers suspect, develops in a latent manner through epigenetic mechanisms. Currently, scientists are still lacking evidence to confirm an unequivocal link between intrafetal epigenetic changes and the consequences of type 2 diabetes, cardiovascular disease, or obesity [86].

#### 3.3.2. Early Nutrition and Maternal Obesity

##### Animal Studies

To date, studies conducted in animal models have identified mechanisms linking early nutrition (such as maternal diet in pregnancy), maternal obesity, and gestational diabetes with cardiovascular disease in offspring [87,88,89,90,91].

Experiments carried out in yellow agouti mouse models showed that the maternal diet during pregnancy affected DNA methylation patterns and the fetus phenotype. Feeding pregnant mice a diet supplemented with methyl group donors resulted in a change in the color of the coat of the offspring from yellow to brown [87]. Dolinoy et al. in their study demonstrated that pregnant mice on a soy-rich diet gave birth to offspring with lower birth weights; this applied to the brown phenotype. Such effects at the metabolic and phenotypic level, according to the authors, may indicate that the soy diet caused epigenetic changes in the fetal life of the offspring, which occurred through methylation [88]. The effects of a high-fat diet on maternal and offspring health were also studied in pregnant mouse models [89]. In pregnant mice fed a high-fat diet, the main effect of this dietary intervention was obesity. On the other hand, in adult offspring, two successive generations were observed to inherit body length and glucose homeostasis. Mice from both generations of offspring had impaired glucose-insulin metabolism, which put them at risk of developing type 2 diabetes. In the last generation, the researchers also noticed changes in the excitatory receptor of growth hormone—the demethylation of certain genes was associated with specific anthropometric traits, according to them [89]. Two studies by Masuyama et al. were also associated with a similar diet intervention. At first, the researchers found that maternal exposure to a high-fat diet during pregnancy causes epigenetic changes in the genes that encode the hormones produced by adipose tissue, adiponectin and leptin, in the offspring [90]. In contrast, another study showed that the more generations are on a high-fat diet (62% fat, type of fats not described) during pregnancy, the stronger the negative effect on each subsequent generation of offspring. A second important observation was that feeding mothers during pregnancy a controlled diet (12% fat, type of fats not described) could partially abolish the effects of the earlier high-fat diet. However, again, in order to observe changes in the external characteristics of the offspring, multigenerational exposure is necessary [91]. Animal model studies on nutritional interventions, maternal obesity, and the effects of these parameters on offspring are presented below (see Table 2).

##### Human Studies

Numerous studies conducted in animal models, even despite efforts to replicate adequate doses for humans, are no substitute for the results of observations and studies involving humans [23,24,25,26,27,28,87,88,89,90,91,92,93].

As is known from studies and clinical practice, newborns of women who begin pregnancy with obesity are often born with macrosomia and have a significantly higher risk of developing obesity in early life, which can worsen in later years of life [23,24,25,26,27,28].

Studies conducted with overweight and obese pregnant women have shown the impact of excessive weight during this period and the pre-pregnancy period on increasing the risk of perinatal complications and outcomes for both mother and child [23,24,25,26,27,28,92,93,94]. The effects of a high maternal pre-pregnancy BMI, maternal overweight or obesity on the baby include: higher risk of macrosomia, higher z-score of subscapular skinfold thickness, higher z-scores of biometry, abdominal area (AA) and abdominal fat mass (AFM) and velocity of estimated fetal weight (EFW), large gestational age (LGA), and inadequate gestational weight gain (GWG), as well as low birth weight [23,24,25,26,92,93,94].

Effects that can affect the baby indirectly, through maternal deterioration, include a higher risk of gestational diabetes, pre-eclampsia, pregnancy-induced hypertension, the need for a cesarean section, and more [24,92,94]. Studies such as The UK Pregnancies Better Eating and Activity Trial (UPBEAT study) and its follow-ups have confirmed the positive effects of dietary intervention and the inclusion of physical activity in overweight and obese women during pregnancy [26,92,93]. Although the study did not confirm statistically significant differences in terms of the risk of developing macrosomia in the child or gestational diabetes in the mother [92]. However, a decrease in glycemic levels occurred only in women in the intervention group [92]. In contrast, in follow-ups to this study, there was a decrease in the z-score of subscapular skinfold thickness in children born to women in the intervention group of the UPBEAT study [93]. In a second study, conducted 3 years after the intervention, Dalrymple et al. indicated a beneficial effect of the intervention on resting heart rate (lower than in the control group) and a slightly lower probability of overweight or obese children [26]. Positive effects were also observed for the development of long-term (3-year) healthy maternal eating habits, which can also potentially promote child nutrition [26].

Despite the many positive reports, all researchers agree on the need for more research and the introduction of similar nutrition interventions in other countries as well, and on a larger scale [23,24,25,26,27,28,92,93,94,95].

Studies with pregnant women on maternal obesity, pre-pregnancy BMI and dietary interventions in overweight or obese women and their effects on offspring are presented below (see Table 3).

#### 3.3.3. Gestational Diabetes Mellitus

Until now, studies in animal models and those in pregnant women have provided a wealth of information on intrafetal exposure to excessive maternal weight [23,24,25,26,87,88,89,90,91,92,93,94]. On the basis of observational studies with humans, researchers have so far discovered several interesting phenomena regarding the mechanisms of impaired carbohydrate metabolism in the fetus [29,96,97]. Based on the aforementioned cohort of The Dutch Hunger Winter, Heijamans et al. made one of the first reports on the connection between epigenetics and type 2 diabetes [96]. The conclusion supported previous theses; according to the researchers, epigenetic changes in the form of DNA methylation underlie the diseases observed in children born after the Dutch famine [96]. Another study in this regard was conducted in the Pima Indian population, more specifically in the offspring of mothers with GDM [97]. The experiment highlighted differences in DNA methylation patterns on promoters associated with genes associated with young adult diabetes (MODY) and T2D [97]. In this sample, disruption of the pathways involved in pancreatic evolution and maintenance of pancreatic islet β-cell sensitivity to glucose and insulin secretion was also observed [97]. The offspring of mothers with GDM had elevated birth body mass index (BMI) scores, as well as a marker such as glycated hemoglobin (HbA1C) [97]. As this was an observational study, the authors were unable to determine whether epigenetics played a primary or secondary role in relation to the formation of offspring phenotypes [97]. The results seem to be sufficiently confirmed to say that maternal hyperglycemia during pregnancy negatively affects the methylation of genes related to pancreatic endocrine functions and increases the risk of developing diabetes in the offspring [97]. In 2012, a meta-analysis that included a systematic review of 13 clinical trials on the association of blood pressure of offspring with maternal gestational diabetes was published. Researchers demonstrated that there was an association between GDM and higher systolic and diastolic blood pressure in the offspring. However, this association was only observed in male offspring; no positive correlation was observed in this regard in girls [29]. There is a growing trend among researchers to emphasize the association of GDM and maternal excess body weight with adverse health changes observed in offspring from an early age [29,96,97]. Increasing numbers of researchers are also emphasizing that adverse intrauterine maturation conditions are related to postnatal conditions such as poor nutrition and sedentary lifestyles and contribute to the development of cardiometabolic diseases [23,24,25,26,92,93,94,95]. At the same time, physicians and scientists unanimously warn that the risk of these diseases is correlated with the number of metabolic disorders detected in pregnant women and with each successive generation [21,27,85,89,90,91].

#### 3.3.4. Gut Microbiota

In recent years, increasing attention has been paid to the utility of the intestinal microbiota, i.e., the totality of microorganisms, predominantly bacteria, that populate the human gastrointestinal tract. The microorganisms that populate our body have many important functions and are responsible, among other things: for the development and proper functioning of the immune system or the production of B and K vitamins [98]. They protect against colonization of the gastrointestinal tract by pathogenic organisms and also influence the broader metabolism and functioning of the entire organism [98,99,100,101].

The development and composition of the gut microbiota of the offspring is influenced by many factors, such as genetics, prenatal factors, maternal health and nutrition, gestational age achieved and type of delivery, as well as how the child is fed after birth [30,31]. This seems to be important also because the gut microbiota stabilizes and reaches a composition approximately 60–70% similar to that observed in adulthood, during the first 3 years of a child’s life [30]. The remainder of the microbiota may change under the influence of the following factors: diet, physical activity and lifestyle, or previous surgical procedures. Factors related to bacterial infections and their treatment with antibiotics or other drugs with bacteriostatic or bactericidal effects also play an important role [30,31,32,33]. Unwavering interest has been shown in proper nutrition and the role that it can play in all stages of life, including during the intrauterine period of the fetus [30]. For a long time, scientists believed that the gastrointestinal tract of the newborn was not colonized by any bacteria and that colonization occurred first during birth and the passage of the baby through the birth canal. However, these assumptions proved to be incorrect and currently available publications indicate the presence of bacteria of the mother’s microbiota in newborns [102,103]. Microbiological studies have shown the presence of mother microorganisms in the meconium of the baby, as well as in the placenta, umbilical cord blood, amniotic fluid, and amniotic membranes [32,33,102,103]. It is assumed that they enter the fetus from the maternal circulation. However, the phenomenon of macrobiotic colonization of the fetus has not yet been thoroughly investigated, and so far its role has been considered negligible. Various sources state that the microbiota is inherited from 2–9% and that it is inherited primarily by the child from the mother and that it passes through the placenta to some extent. Its migration from the mother to the fetus occurs via the bloodstream [102]. Therefore, changes in the mother’s microbiota, including those occurring during pregnancy, pre-partum weight, prevalence of GDM, diet during pregnancy and use of antibiotic therapy, appear to be important [31,104].

## 4. Limitations and Future Research

A limitation of this review may certainly be the citation of studies in animal models, since we know from many other studies that often even the most similar doses of substances may not produce the same or even similar effects in humans. This is because numerous metabolic pathways in the bodies of humans and rodents operate differently on many levels. Many of the pathways identified in animal models have also not yet been studied in human studies, or may never be studied for ethical reasons.

As authors, we made every effort to ensure that the studies we collected were as objective as possible, as may be indicated by, among other things, the large number of studies cited, clear inclusion and exclusion criteria for studies in the review, and the reporting of all study results, not just those supporting our thesis. However, despite the authors’ efforts, a narrative review is a form of scientific work that may carry a greater risk of subjective evaluation than other forms of review work, such as a systematic review or meta-analysis. The systematic review is considered to be the gold standard of evidence synthesis, but also carries the potential disadvantages of narrow scope, and requiring more time and resources to prepare and update. Meta-analysis, on the other hand, requires similar research criteria and conditions, but most importantly, the existence of multiple scientific studies on the same issue, with each individual study presenting measurements subject to some degree of statistical error.

In future studies, there is a need for long-term monitoring of the offspring of women subjected to nutritional or behavioral interventions during preconception and pregnancy. Additionally, it is important that future studies be multi-center studies conducted with women and their offspring from different ethnic and social races to exclude the influence of distracting factors.

## 5. Conclusions

The fact is that the prevalence of overweight and obesity is increasing in children and in all other age categories. At the same time, cardiovascular disease associated with these conditions continues to be the leading cause of death worldwide. In contrast, it is known that metabolic diseases, including obesity or CVD, are also hugely related to environmental factors. In light of current research findings on the impact of the preconception period, among other things, the significant role of maternal BMI before pregnancy and the prenatal period on the future health of the child, it seems reasonable to ensure that women are adequately prepared for this role. Our goal is not to hold the mother completely responsible for her child’s metabolic health. Instead, we want to point out that preparing for pregnancy, ensuring proper weight, nutrition and physical activity are very important issues reflected in the future health of the child. Among other things, the aforementioned global nutrition education programs supporting women during the preconception period, pregnancy, the postpartum period and the first years of a child’s life can be helpful in this regard. In their interventions, researchers should pay special attention to the group of pregnant women who have failed to deal with overweight or obesity before pregnancy. Care should be taken to include these women in programs that can help introduce a healthy diet or incorporate daily physical activity. Such measures can yield beneficial results for both the mother’s and child’s health, whether in the early stages of life or in adulthood. However, more research is needed, especially to examine the relationship between a healthy diet and the weight of mothers and the metabolic results of their offspring.

## Figures and Tables

**Table 1:** Eligibility Criteria for prenatal factors analysis.

Inclusion Criteria	Exclusion Criteria
Articles in English Nutritional programming and prenatal issues Nutritional programming and the preconception period Meta-analyses Systematic reviews Randomized clinical trials Observational studies Animal studies Historical data	Articles in a language other than English Nutritional programming and postnatal issues (breastfeeding, protein intake) In vitro studies Book Narrative reviews Review of reviews Research with pregnant adolescent (<16 years old)

**Table 2:** Animal studies on dietary interventions during pregnancy, maternal obesity, and its impact on offspring.

Authors and Year of Publication	Factor under Examination	Size of the Study Group	Results and Conclusions
Waterland et al., 2003 [87]	Diet supplemented with methyl group donors	Mothers/offspring: *n* = 9/30, unsupplemented group; *n* = 10/39, supplemented group	Feeding pregnant mice a diet supplemented with methyl group donors resulted in a change in the color of the coat of the offspring from yellow to brown. The results of this study may suggest that dietary supplementation may have unintended deleterious effects on the establishment of epigenetic gene regulation in humans.
Dolinoy et al., 2006 [88]	Maternal genistein- supplemented diet of mice during gestation	Mothers/offspring: *n* = 15/52, unsupplemented group; *n* = 12/44, genistein-supplemented	The study reports that maternal supplementation of mice with genistein during gestation, at levels comparable to humans consuming a high-sugar diet, changed the coat color of heterozygous viable yellow agouti (A(vy/a) offspring toward pseudoagouti. The extent of this DNA methylation was similar in different tissues (endodermal, mesodermal, and ectodermal), indicating that genistein acts during early embryonic development. Furthermore, genistein-induced hypermethylation persisted into adulthood, reducing ectopic Agouti expression and protecting offspring from obesity.
Dunn et al., 2009 [89]	The 4.73-kcal/g high-fat diet consists of (by kcal): 7% corn starch, 10% maltodextrin, 17% sucrose, 39% lard, 20% casein, 0.3% l-cystine, 6% soybean oil, and essential vitamins and minerals. The 4.00-kcal/g house chow diet consisted of 28% protein, 12% fat, and 60% carbohydrate.	Mothers/first-generation offspring: *n* = 20/128, high-fat-fed (mHF); *n* = 12/65, chow-fed (mCH)	The study indicates that due to maternal exposure to a high-fat diet, the effect of a significant increase in body length persisted in the offspring for at least two generations. This phenotype is not attributed to altered intrauterine conditions or maternal dietary behaviour, as maternal and paternal lines were able to pass on the effect, supporting a true epigenetic mode of inheritance. A heritable trait of reduced insulin sensitivity was also detected between two generations. These studies suggest that the heritability of body length and glucose homeostasis is modulated by maternal diet over multiple generations, providing a mechanism in which length can increase rapidly in concert with caloric availability.
Masuyama et al., 2012 [90]	Maternal high-fat diet (HFD): energy content of 62% fat, 18% protein, and 20% carbohydrate. Maternal and offspring control diet (CD): 12% fat, 28% protein, and 60% carbohydrate.	Mothers/offspring: *n* = 6/24, offsprings from dams exposed to a high-fat diet during pregnancy (OH); *n* = 6/24, offsping from dams exposed to a conrtolled diet during pregnancy (OC)	OH mice were significantly heavier than OC mice. OH mice had higher blood pressure and worse glucose tolerance than OC mice. Total triglyceride and leptin levels were significantly higher, and adiponectin levels were significantly lower in OH mice compared to OC mice. This was due to changes in leptin and adiponectin expression in white adipose tissue. The study suggests that exposure to a high-fat diet in utero may cause a metabolic syndrome-like phenomenon through epigenetic modifications of adipocytokine, adiponectin and leptin gene expression.
Masuyama et al., 2015 [91]	High-fat diet 4 weeks before and during pregnancy and control diet (diet composition assumptions as Masuyama et al., 2012).	Mothers/offspring: differentiated according to the number of generations studied (group F0–2 or group F0–4).	The effect of maternal HFD on offspring over many generations in metabolic syndrome-like phenomena, such as weight gain and fat mass gain, glucose intolerance, hypertriglyceridemia, abnormal levels of adiponectin and leptin, and hypertension, was cumulative from expression and epigenetic changes in the genes of the aforementioned hormones. The controlled diet during pregnancy in subsequent generations after HFD in utero exposure decreased and the control diet during pregnancy for 3 generations completely abolished the effects of HFD in utero on body weight and fat mass gain, insulin resistance, serum triglyceride levels, adiponectin and leptin, with epigenetic changes in the adiponectin and leptin genes.

**Table 3:** Human studies on maternal obesity or pre-pregnancy body mass and its effects on offspring.

Authors and year of Publication	Type of Research	Factor under Examination	Characteristic of the Study Group	Results and Conclusions
Johnson et al., 1992 [23]	Longitudal restrospective study	The impact of increased maternal weight before pregnancy and increased weight gain during pregnancy affect pregnancy outcomes.	*n* = 3191 (BMI < 19.8, *n* = 755; BMI = 19.8–26, *n* = 1621; BMI = 27–29, *n* = 329; BMI > 29, *n* = 486) Mean age—data not available	Increased maternal weight before pregnancy (BMI) and increased weight gain during pregnancy were associated with increased risk of fetal macrosomia, birth abnormalities including unplanned cesarean section, postmaturity, though also with reduced incidence of low birth weight. Increased maternal weight gain during pregnancy results in a higher incidence of fetal macrosomia, which in turn leads to increased rates of cesarean section and other serious maternal and fetal complications.
Jensen et al., 2003 [24]	Cohort study	The relationship between pregnancy outcome and prepregnancy overweight or obesity in women with normoclycemia	*n* = 2459 (BMI = 18.5–24.9, *n* = 1094; BMI = 25.0–29.9, *n* = 728; BMI *≥* 30.0, *n* = 637) Age (mean ± SD) = 30.2 ± 5.1 years	Compared with normal-weight women (BMI 18.5–24.9 kg/m^2^), the risk of complications (preeclampsia, pregnancy-induced hypertension, cesarean section, induction of labor and macrosomia) was significantly increased in both overweight (BMI 25.0–29.9 kg/m^2^) and obese women (BMI ≥ 30.0 kg/m^2^). Pre-pregnancy overweight and obesity are associated with adverse pregnancy outcomes in normoglycemic women.
Poston et al. 2015 [92]	RCT	“The UK Pregnancies Better Eating and Activity Trial (UPBEAT study)” The relationship between an intervention including diet and physical activity and a reduction in the incidence of gestational diabetes and macrosomia in infants.	*n* = 1553 (Intervention group: *n* = 783, maternal age (mean ± SD) = 30.5 ± 5.5 years, BMI (mean ± SD) = 36.3 ± 5.0 kg/m^2^); Control group *n* = 772, maternal age (mean ± SD) = 30.4 ± 5.6 years, BMI (mean ± SD) = 36.3 ± 4.6 kg/m^2^)	Gestational diabetes was found in 160 (25%) women in the intervention group and in 172 (26%) women in the control group (*p* = 0.68). Macrosomia was found in 71 (9%) infants in the intervention group, compared to 61 (8%) infants in the control group (*p* = 0.40). Thus, there were no statistically significant differences in this regard. In contrast, there was a decrease in glycemia in the intervention group of women. Behavioral intervention including dietary change (promote a healthy pattern of eating but not to restrict energy intake) and physical activity in women with obesity during pregnancy is insufficient. There were no statistically significant differences in preventing gestational diabetes and reducing the incidence of infants with macrosomia between the intervention and control groups.
Patel et al., 2017 [93]	RCT	Postnatal follow up for a RCT ‘The UPBEAT study”. The impact of antenatal lifestyle intervention, including improvements in maternal diet and reduced gestational weight gain (GWG) in obese pregnant women affects reduction in infant adiposity	*n* = 698 (Intervention group: *n* = 342, maternal age (mean ± SD) = 31.3 ± 5.04 years, BMI (mean ± SD) = 36.17 ± 4.98 kg/m^2^); Control group *n* = 356, maternal age (mean ± SD) = 31.0 ± 5.58 years, BMI (mean ± SD) = 36.31 ± 4.69 kg/m^2^)	There were no differences in the z-score of triceps skinfold thickness between the intervention and standard care arms, though the z-score of subscapular skinfold thickness was lower in the intervention arm. Analysis of the data may indicate that the lower subscapular skinfold thickness in the infants was due to changes in the mother’s prenatal diet and weight gain during pregnancy, rather than postpartum diet.
O’Brien et al., 2020 [25]	RCT	The relationship of fetal growth velocity and fetal obesity under maternal obesity	*n* = 911 (BMI = 25.0–29.9, *n* = 376; BMI = 30.0–34.9, *n* = 271; BMI = 35.0–39.9, *n* = 153; BMI *≥* 40.0, *n* = 111) Age (mean) = 29.6 years	The fetus of women with giant obesity (BMI ≥ 40.0) showed the greatest increases in all z-scores of biometry, abdominal area (AA) and abdominal fat mass (AFM) compared to women classified as overweight (BMI 25.0–29.9). Maternal obesity was associated with an increase in all parameters, including: AFM and the velocity of estimated fetal weight (EFW) over time. Women with class 3 obesity (BMI ≥ 40.0) may be at increased risk of perpetuating intergenerational transmission of obesity to their offspring.
Sun et al., 2020 [94]	Clinical trial	The possible impact of pre-pregnancy BMI and gestational weight gain (GWG) on pregnancy outcomes and maternal and infant complications.	*n* = 3172 (BMI < 18.5, *n* = 420, BMI = 18.5–24.9, *n* = 2292; BMI = 25–29.9, *n* = 401, BMI *≥* 30.0, *n* = 59)	Overweight and inadequate GWG were risk factors for gestational diabetes mellitus (GDM) and large gestational age (LGA), and inadequate GWG was a risk factor for low birth weight. Overweight and obesity were risk factors for gestational hypertension, macrosomia, among others. Both overweight and obesity before pregnancy and excessive GWG are associated with a higher risk of developing GDM, GHp, macrosomia and LGA.
Dalrymple et al., 2021 [26]	RCT	The impact of behavioral intervention (“The UPBEAT study”) on cardiometabolic outcomes in children (and on sustained improvements in maternal lifestyle over 3 years postpartum)	Three-year-old children: *n* = 514: Intervention group: *n* = 250; Control group: *n* = 264.	There were no differences between the children of mothers in the intervention and control groups regarding the thickness of the subscapular skinfold. However, children of mothers in the intervention group had lower resting heart rates and a non-significantly lower likelihood of being overweight/obese. The study indicated that prenatal dietary and physical activity intervention in obese women is associated with lower offspring heart rates, as well as sustained improvements in maternal diet. However, the researchers point to the need to verify the study’s findings in future cohorts.

BMI—body mass index; RCT—randomized controlled trial; SD—standard deviation.

## Data Availability

Not applicable.
